# Supplementary material for: Non-viral DNA delivery and TALEN editing correct the sickle cell mutation in hematopoietic stem cells
Source: Nat Commun. 2024 Jun 11;15:4965. doi: 10.1038/s41467-024-49353-3 (PMC11166989; doi:10.1038/s41467-024-49353-3)
Supplement: Supplementary file 1 — Supplementary Information [file 41467_2024_49353_MOESM1_ESM.pdf]

Supplementary materials

Non-viral DNA delivery coupled to TALEN gene editing efficiently corrects the sickle cell mutation in long-term HSCs

Arianna Moiani[1*], Gil Letort[1], Sabrina Lizot[1], Anne Chalumeau[2], Chloe Foray[1], Tristan Felix[2], Diane Le Clerre[1], Sonal Temburni-Blake[3], Patrick Hong[3], Sophie Leduc[1], Noemie Pinard[1], Alan Marechal[1], Eduardo Seclen[3], Alex Boyne[3], Louisa Mayer[3], Robert Hong[3], Sylvain Pulicani[1], Roman Galetto[1], Agnès Gouble[1], Marina Cavazzana[4,5,6], Alexandre Juillerat[3], Annarita Miccio[2], Aymeric Duclert[1], Philippe Duchateau[1], Julien Valton[1*]

1. Cellectis S.A., 8 Rue de la Croix Jarry, 75013 Paris, France.
2. Université Paris Cité, Imagine Institute, Laboratory of Chromatin and Gene Regulation During Development, INSERM UMR 1163, Paris, France.
3. Cellectis, Inc., 430 East 29th Street, New York, NY 10016, USA.
4. Biotherapy Clinical Investigation Center, Necker Children's Hospital, Assistance Publique Hopitaux de Paris, Paris, France
5. Human Lymphohematopoiesis Laboratory, Imagine Institute, INSERM UMR1163, Paris Cité University, Paris, France
6. Biotherapy Department, Necker Children's Hospital, Assistance Publique Hopitaux de Paris, Paris, France
*Corresponding authors: arianna.moiani@cellectis.com, julien.valton@cellectis.com

Table of contents

-**Supplementary Fig. 1**. Schematic for *HBB* gene correction strategies using TALEN coupled with viral or non-viral DNA delivery strategies.

-**Supplementary Fig. 2.** Optimization of TALEN gene correction protocol at the *HBB* locus in HSPCs mobilized from healthy donors in R&D and GMP-compatible conditions.

-**Supplementary Fig. 3.** Single-cell RNAseq characterization of the different editing protocols in plerixafor-mobilized HSPCs before *in vivo* injection.

-**Supplementary Fig. 4.** Correction of the sickle phenotype in erythroid cells differentiated from edited HSPCs.

-**Supplementary Fig. 5.** TALEN off-target sites analysis and genomic rearrangements after editing at HBB locus.

-**Supplementary Fig. 6.** Characterization of TALEN-mediated genomic rearrangements after optimized viral editing at *HBB* locus in HbSS HSPCs.

- **Supplementary Fig 7.** Characterization of TALEN-mediated genomic adverse events occurring at *p53* after viral and non-viral editing at HBB locus of HSPCs in GMP compliant conditions.

-**Supplementary Fig. 8.** FCM analysis strategy for assessing *in vitro* CD34+ Viability and Purity.

-**Supplementary Fig. 9.** FCM analysis strategy for assessing *in vitro* erythroid differentiation of non-mobilized HSPCs derived from HbSS patients.

-**Supplementary Fig. 10.** FCM analysis strategy for dissecting the engrafted human population in the bone marrow of NCG and NBSGW mice.

-**Supplementary Fig. 11.** FCM analysis strategy for dissecting the multilineage engraftment in the bone marrow of NCG and NBSGW mice.

Supplementary Figures

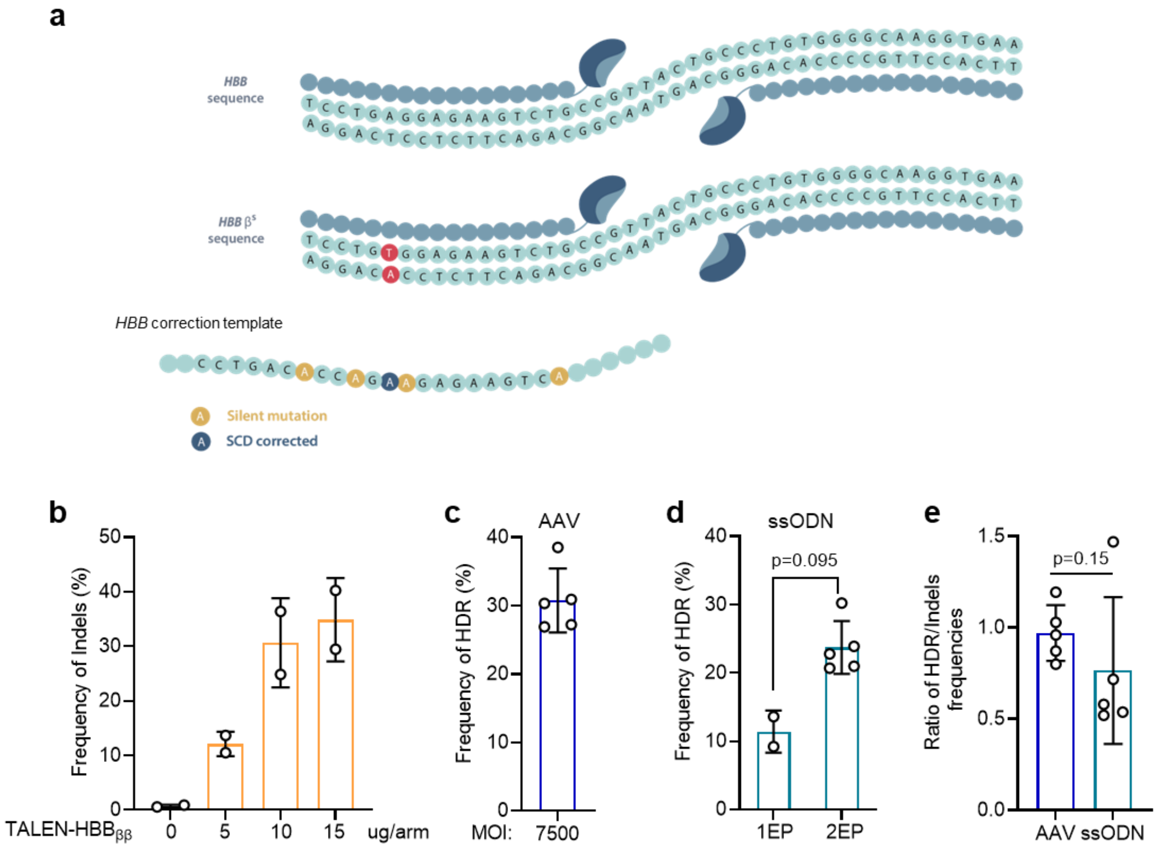

Supplementary Fig. 1: Schematic for *HBB* gene correction strategies using TALEN coupled with viral or non-viral DNA delivery strategies. a Representative schema and sequences of TALEN binding at the HBB locus and HBB gene correction template. SCD mutation in the TALEN binding site is highlighted in red. Silent mutations and SCD correction are indicated in different colors in the HBB correction template. **b** Frequencies of indels obtained by high-throughput DNA sequencing of TALEN-HBB$_{\beta\beta}$ at increasing doses (mRNA conc. per arm). Data represents mean ± SD, for n = 2 independent biological replicates. **c** Frequencies of HDR obtained by high-throughput DNA sequencing or ddPCR of TALEN-HBB$_{\beta\beta}$ coupled with AAV transduction at a selected multiplicity of infection (MOI). Data represents mean ± SD, for n = 5 independent biological replicates. **d** Frequencies of HDR obtained by ddPCR of TALEN-HBB$_{\beta\beta}$ coupled with ssODN delivered in co-transfection with TALEN (1EP) or in a second electroporation (2EP). Data represents mean ± SD, for n = 2 (1EP) and 5 (2EP) independent biological replicates. Mann-Whitney two-tailed unpaired non-parametric test. *P*-value are indicated. **e** Ratio of HDR/Indel frequencies obtained from the

selected protocol for AAV or ssODN mediated editing. Data represents mean ± SD, for $n = 5$ independent biological replicates. A non-parametric, two tailed, unpaired Mann-Whitney test was used for statistical analysis of the data. P-value are indicated. Source data are provided as a Source Data file.

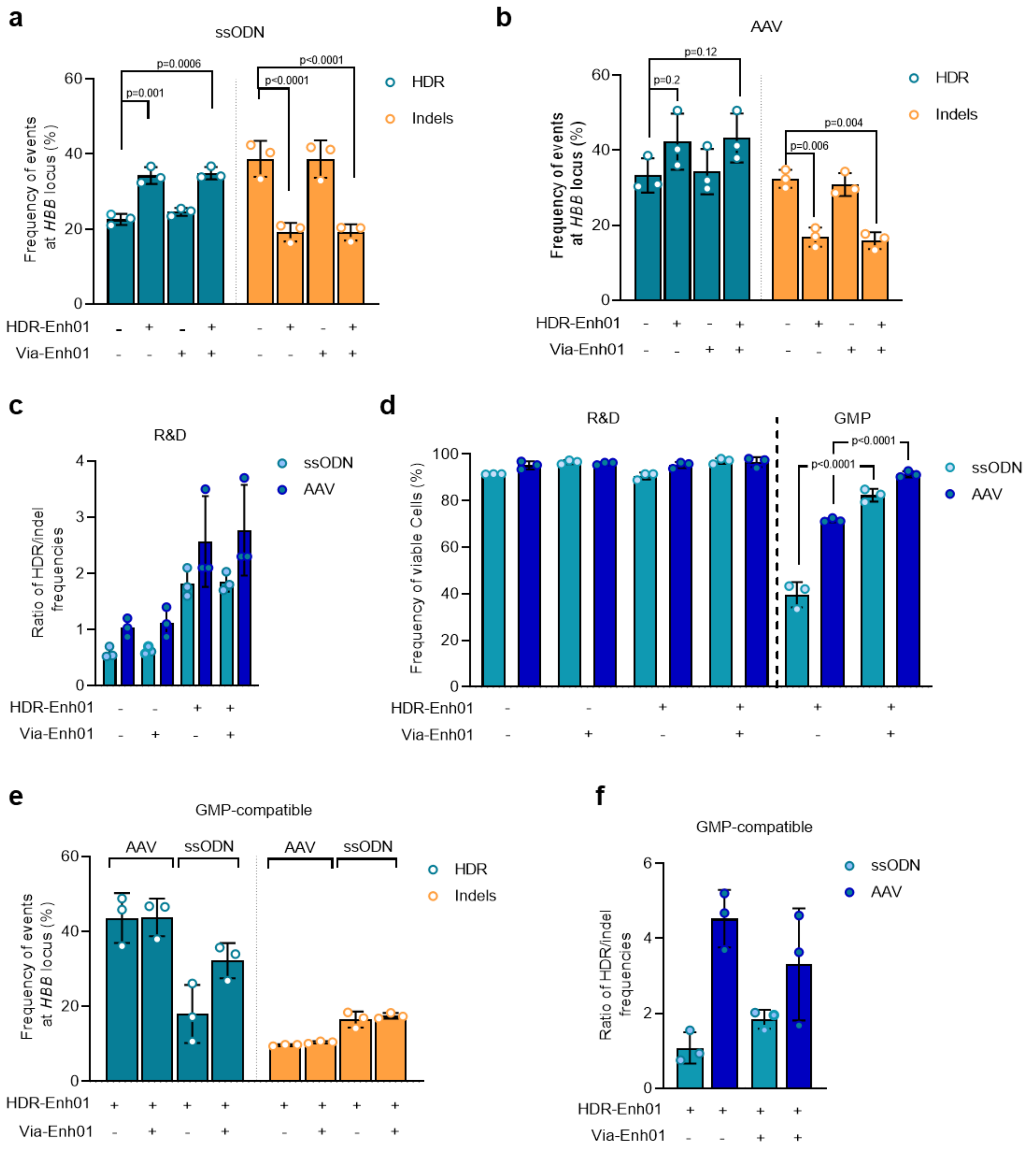

Supplementary Fig. 2: Optimization of TALEN gene correction protocol at the *HBB* locus in HSPCs mobilized from healthy donors in R&D and GMP-compatible conditions. Frequency of homology-directed repair (HDR) and insertion/deletion (Indels) allelic events at *HBB* locus measured at day 4 by ddPCR in mobilized HSPCs edited with TALEN coupled with

ssODN **(a)** or AAV **(b)** in presence (+) or absence (-) of HDR-Enh01 and/or Via-Enh01. Data are expressed as mean ± SD, for *n* = 3 biologically independent experiments and donors. A two-way ANOVA followed by Bonferroni multi-comparison test was used for statistical analysis of the data. *P*-values are indicated. **c** Ratio of HDR/indels frequencies obtained in ssODN or AAV edited HSPCs in presence (+) or absence (-) of HDR-Enh01 and/or Via-Enh01. Data are expressed as mean ± SD, for n = 3 biologically independent experiments and donors. **d** Frequencies of viable cells evaluated at day 4 by flow cytometry in ssODN or AAV edited cells in R&D or GMP compatible conditions in presence (+) or absence (-) of HDR-Enh01 and/or Via-Enh01. Data are expressed as mean ± SD, for *n* = 3 biologically independent experiments and donors. A two-way ANOVA followed by Bonferroni multi-comparison test was used for statistical analysis of the data. *P*-value are indicated. **e** Frequency of homology-directed repair (HDR) and insertion/deletion (Indels) allelic events at *HBB* locus measured at day 4 by ddPCR in mobilized HSPCs edited with TALEN coupled with ssODN or AAV in presence (+) or absence (-) of HDR-Enh01 and/or Via-Enh01 in GMP-compatible conditions. Data are expressed as mean ± SD, for n = 3 biologically independent experiments and donors. **f** Ratio of HDR/indels frequencies obtained in ssODN or AAV edited HSPCs in presence (+) or absence (-) of HDR-Enh01 and/or Via-Enh01 in GMP-compatible conditions. Data are expressed as mean ± SD, for n = 3 biologically independent experiments and donors. Source data are provided as a Source Data file.

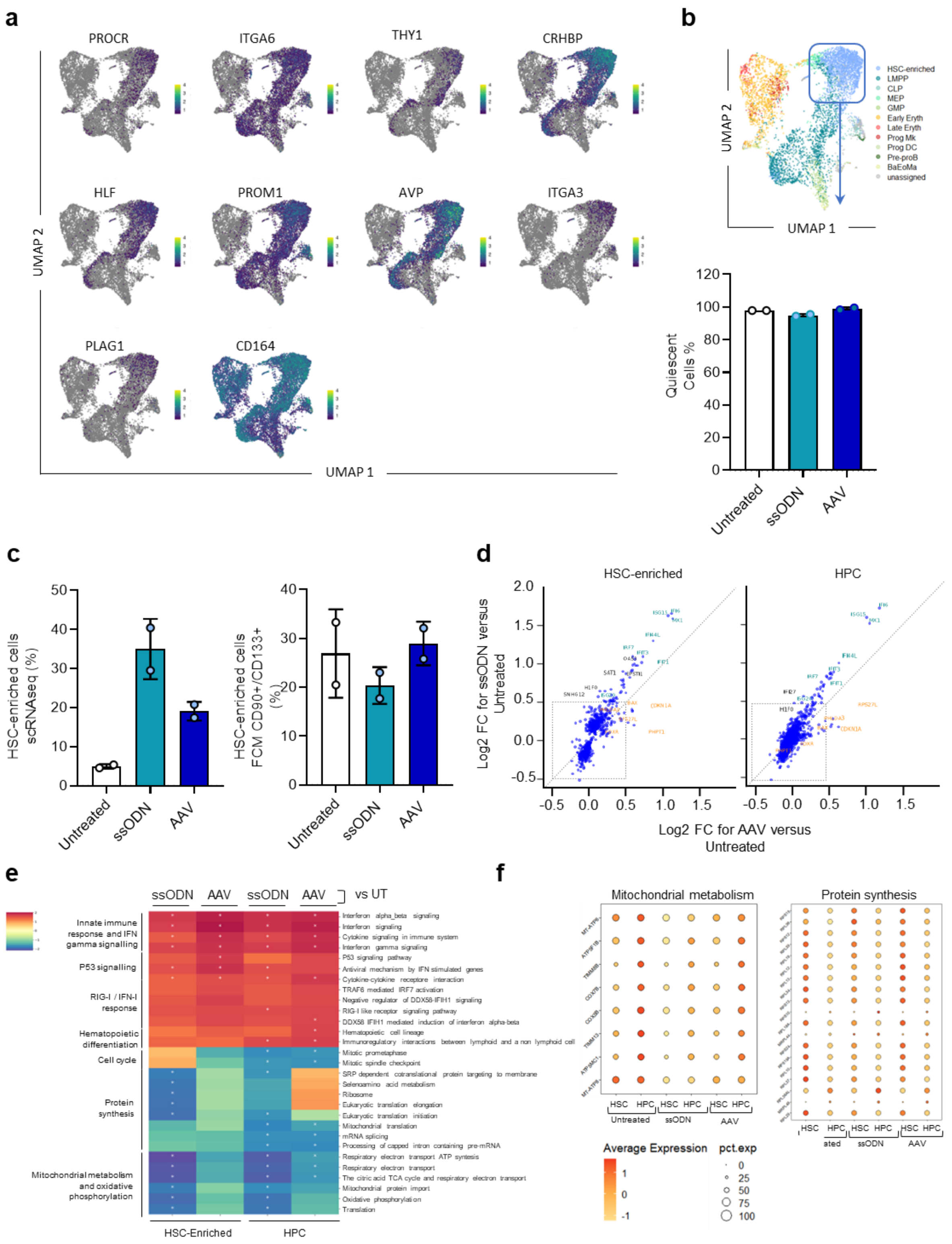

Supplementary Fig. 3: Single-cell RNAseq characterization of the different editing protocols in plerixafor-mobilized HSPCs before *in vivo* injection. a UMAP (Uniform Manifold Approximation and Projection) plot comprising 5'scRNAseq data from HSPCs the day of *in vivo* injection. Normalized expression of selected mRNAs associated with primitive hematopoietic stem cell profiles are represented. The color of each dot indicates the average

expression of the gene according to the scale shown. **b** UMAP plot representing clusters and associated cell types as defined by Azimuth and indicated by name and colors. Blue box and arrow indicate the selected subpopulation (hematopoietic stem cell (HSC)-enriched) selected to analyze the frequency of quiescent cells represented in the right plot and evaluated in ssODN-edited and AAV-edited HSPCs compared to untreated cells. Data represent mean ± SD, for n = 2 donors. **c** Frequency of HSC-enriched population defined by scRNAseq data (left plot) or defined by flow cytometry (right plot) assessed in ssODN-edited and AAV-edited HSPCs compared to untreated cells. **d** ScatterPlot showing differentially expressed genes (DEG) in gene-edited HSC or Hematopoietic Progenitor Cells (HPCs) compared to untreated samples. Each dot represents log2FC for each DEG in ssODN (y-axis) or AAV (x-axis) edited samples. Top deregulated genes in the ssODN and AAV groups are shown in blue and orange, respectively. **e** Heatmap showing Normalized enrichment score (NES) for GSEA performed for the HSC-enriched or HPC population. All DEGs obtained from edited cells were ranked by Log2FC value after comparison to Untreated control. *p-adjusted < 0.01. **f** DotPlots from scRNAseq data representing gene expression profiles for selected relevant genes belonging to mitochondrial metabolism or protein synthesis (rows) among HSC and the rest of HPC from Untreated, ssODN- and AAV-edited cell groups (columns). The size of each dot represents the percentage of cells expressing a specific gene. The color of each dot indicates the average expression of the gene according to the scale shown.

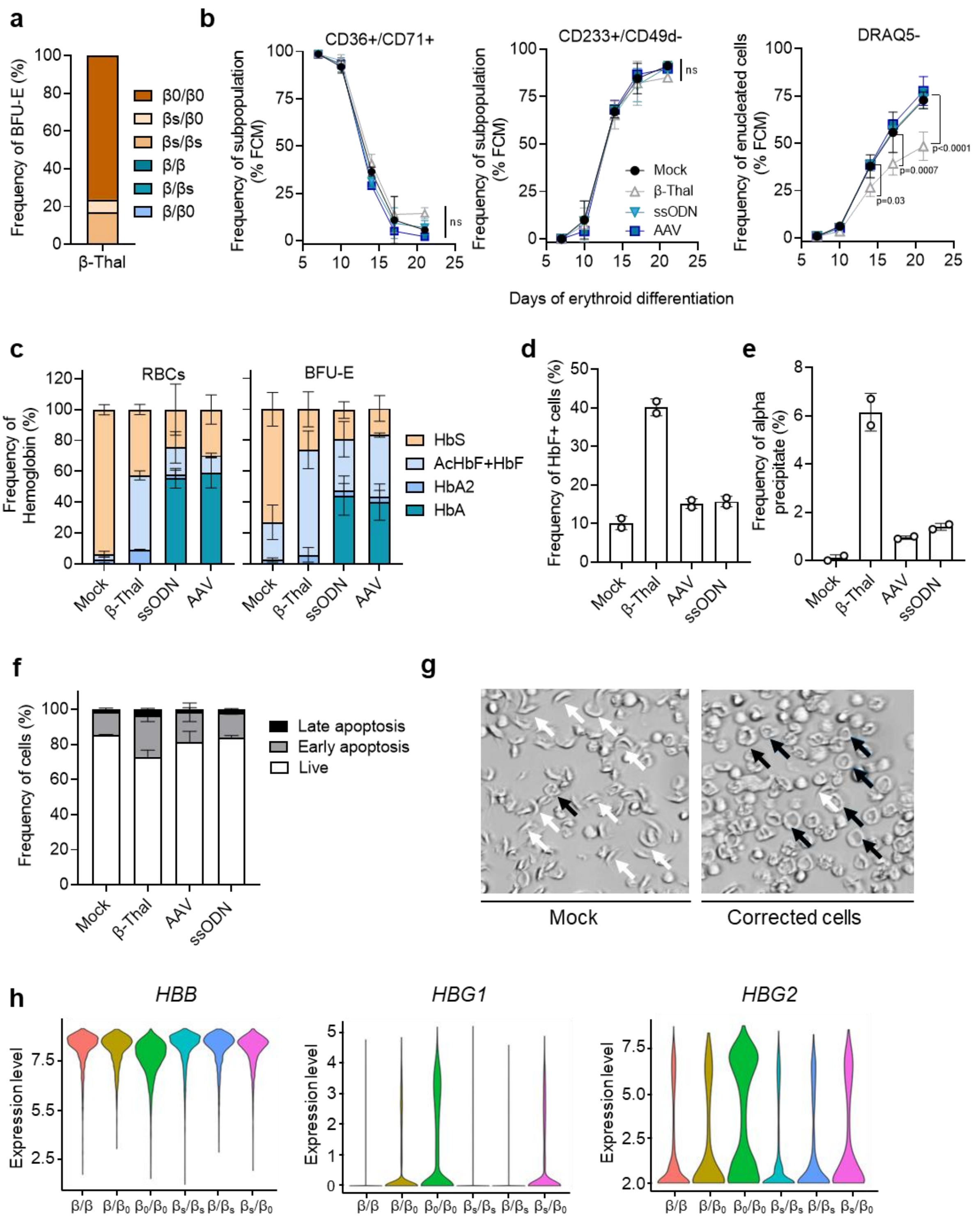

Supplementary Fig. 4: Correction of the sickle phenotype in erythroid cells differentiated from edited HSPCs. a Frequency of single BFU-E colonies harboring each bi-allelic genotype as assigned by ddPCR data for β-Thal control sample. β= corrected allele, β_s = sickle allele, β^0= Indel allele. **b** Proportions of cells expressing selected markers CD36+/CD71+ (left plot), CD233+/CD49d- (center plot) indicating the efficiency of erythroid differentiation and enucleation (DRAQ5-, right plot) over time for ssODN-edited and AAV-edited cells compared

to Mock and β-Thal controls. Data are expressed as mean ± SD, for n=3. Two-way ANOVA followed by Bonferroni multi-comparison test. *P*-values are indicated. CE-HPLC quantification of globin tetramers in erythroid cells **(c, left panel)** or BFU-E colonies **(c, right panel)** differentiated from non-mobilized HSPCs from HbSS patients corrected with ssODN or AAV and compared to Mock and β-Thal controls. Data are expressed as mean ± SD, for n=3 independent biological experiments and donors. **d** Frequency of fetal globin (HbF) + cells evaluated by flow cytometry in ssODN and AAV edited cells compared to Mock and β-Thal controls. Data are expressed as mean ± SD, for n=2 independent biological experiments and donors. **e** Frequency of alpha precipitates assessed by CE-HPLC in erythroid cells differentiated from HbSS patients corrected with ssODN or AAV and compared to Mock and β-Thal controls. Data are expressed as mean ± SD, for n=2 independent biological experiments and donors. **f** Frequency of live, early apoptotic, and late apoptotic subsets in erythroid cells differentiated from HbSS patients corrected with ssODN or AAV and compared to Mock and β-Thal controls. **g** *In vitro* sickling assay measuring the proportion of sickle RBCs. Representative photomicrographs of RBCs derived from Mock or corrected samples. Light white arrows indicate sickling cells, and black arrows indicate normal cells. **h** Violin Plots from scRNAseq data representing gene expression levels for *HBB*, *HBG1*, and *HBG2* evaluated in cells harboring a specific bi-allelic genotype shown in x-axis and assessed from mRNA. β= corrected allele, β_s = sickle allele, β_0= Indel allele. Source data are provided as a Source Data file.

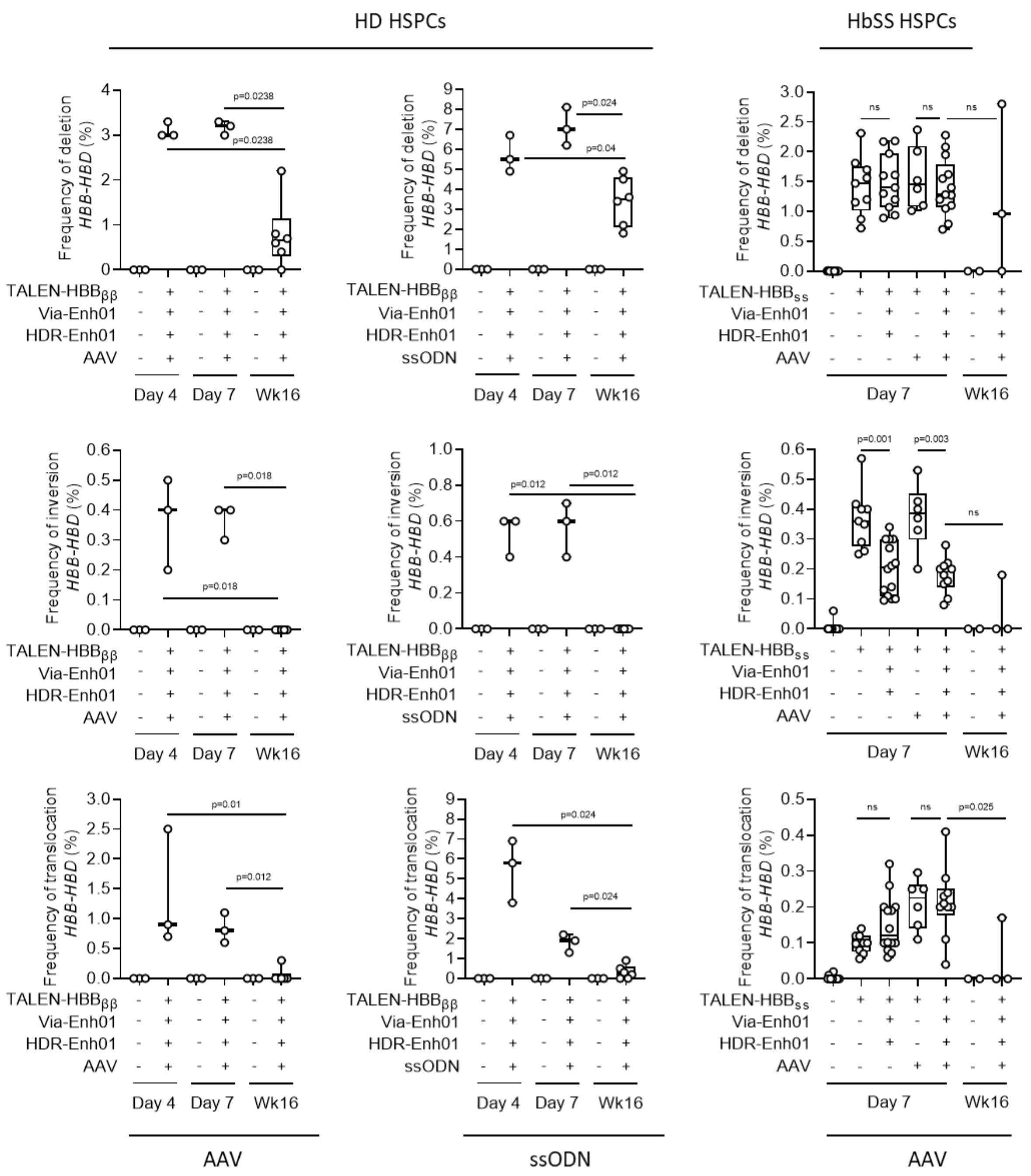

Supplementary Fig. 5: TALEN off-target sites analysis and genomic rearrangements after editing at HBB locus. Frequencies of deletions (upper panels), inversions (middle panels), translocations (bottom panels) at the HBB-HBD locus assessed by ddPCR in ssODN or AAV edited HSPCs derived from healthy donor (HD) or HbSS patients (HbSS) at day 4 (D4), day 7 (D7) of culture and 16 weeks after NCG engraftment onset, in hCD45+ bone-marrow-engrafted-cells (WK16). On each box plot, the central mark indicates the median value, the bottom and top edges of the box indicate the interquartile range (IQR), and the whiskers represent the maximum and minimum data point. Each point represents one experiment, performed with a given HD or HbSS donor in technical simplicate. A non-parametric, two tailed

and unpaired Mann-Whitney test was used for statistical analysis of the data. *P*-value are indicated. Source data are provided as a Source Data file.

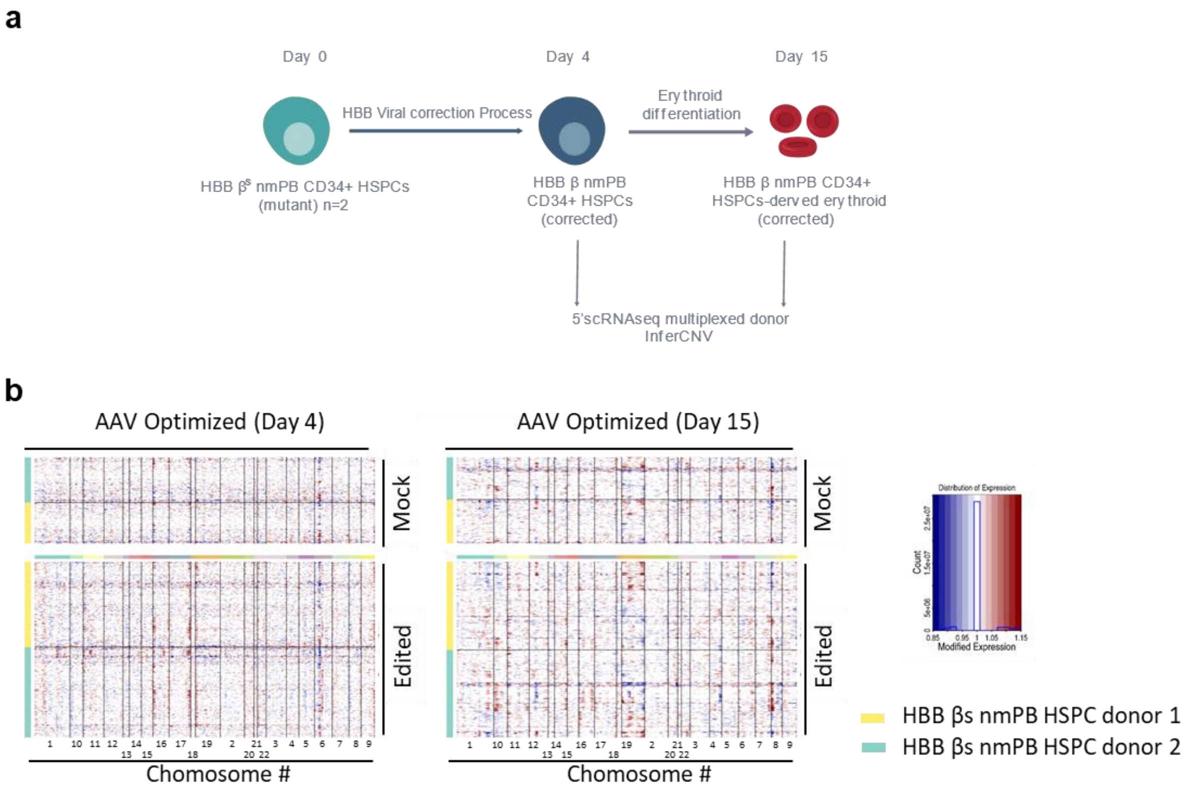

Supplementary Fig. 6: Characterization of TALEN-mediated genomic adverse events after optimized viral editing at *HBB* locus in HbSS HSPCs. **a** Representative schema of scRNAseq InferCNV to identify the potential large genomic rearrangement including gain and loss of heterozygosity in HSPCs, n=2 HbSS donors transfected with mRNA encoding TALEN-HBB_{SS}, Via-Enh01, HDR-Enh01 and transduced with AAV. **b** scRNAseq InferCNV results obtained from samples gathered 4 days post HSPC editing (Day 4) and after editing and erythroid differentiation (Day15). Each individual row corresponds to a single cell and each column corresponds to a specific gene and its genomic position, grouped by chromosome. Red color represents increase in gene expression, while blue color represents decrease in gene expression.

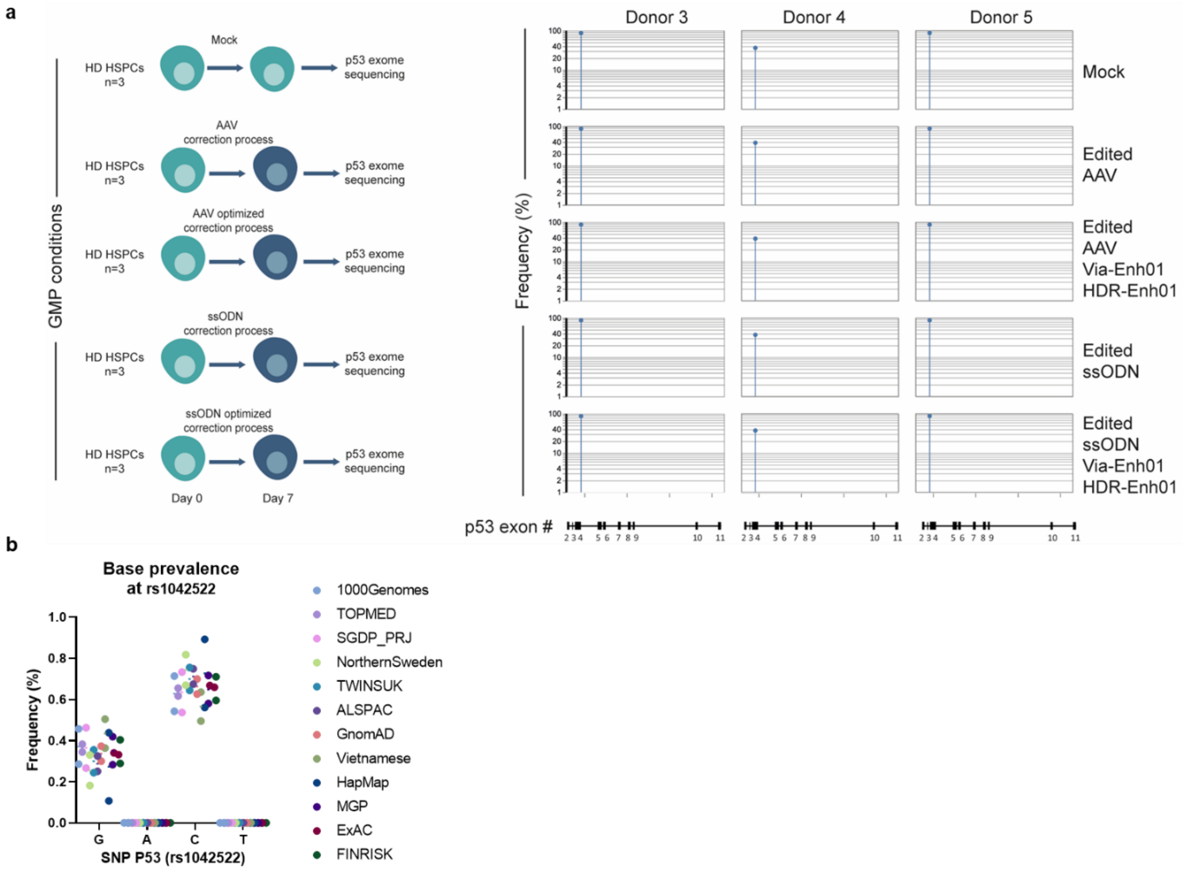

Supplementary Fig. 7: Characterization of TALEN-mediated genomic adverse events occurring at *p53* after viral and non-viral editing at HBB locus of HSPCs in GMP compliant conditions. **a** *p53* exome sequencing obtained from the genomic DNA of Mock HD HSPCs or of HD HSPCs edited in GMP conditions by the viral and non-viral gene editing process in the presence or absence of HDR-Enh01 and Via-Enh01. Mock and edited cells were gathered 7 days post-editing, and their genomic DNA was analyzed using a *p53*-specific exome sequencing. The frequency and exonic position of *p53* variants obtained in each donor (n=3 independent biological experiments and donors) and in each experimental group are indicated. The detection threshold was set to 1% according to the *p53* exon sequencing kit's manufacturer guidelines. **b** The G->C *p53* variant identified in the *p53* exon 4 of the Mock and edited experimental groups of donors 3, 4 and 5 corresponds to a natural single nucleotide variant frequently identified in healthy donors (rs1042522, worldwide median prevalence of 66.4%, one dot corresponds to the prevalence obtained in one SNP database; database names are indicated in the legend).

CD34+ viability and purity

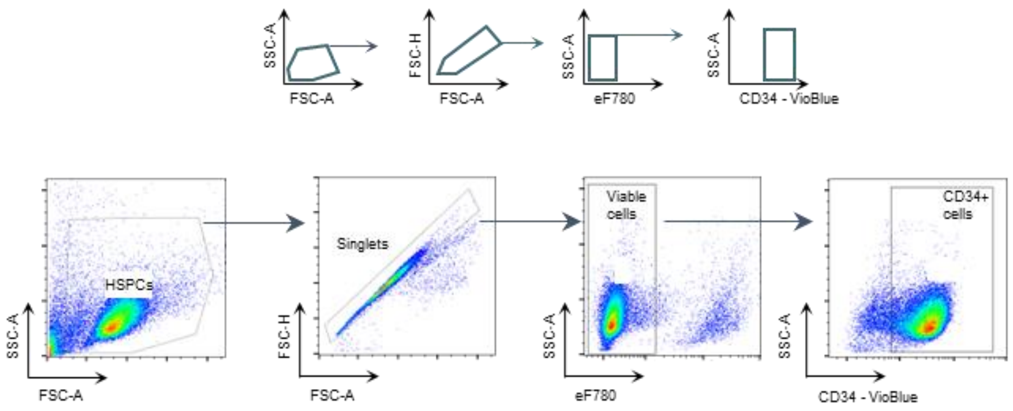

Supplementary Fig. 8: FCM analysis strategy for assessing *in vitro* CD34+ Viability and Purity. FCM gating strategy for assessing the viability and purity of edited CD34+ HSPCs with representative FCM plots and analysis showing viable and CD34+ cells.

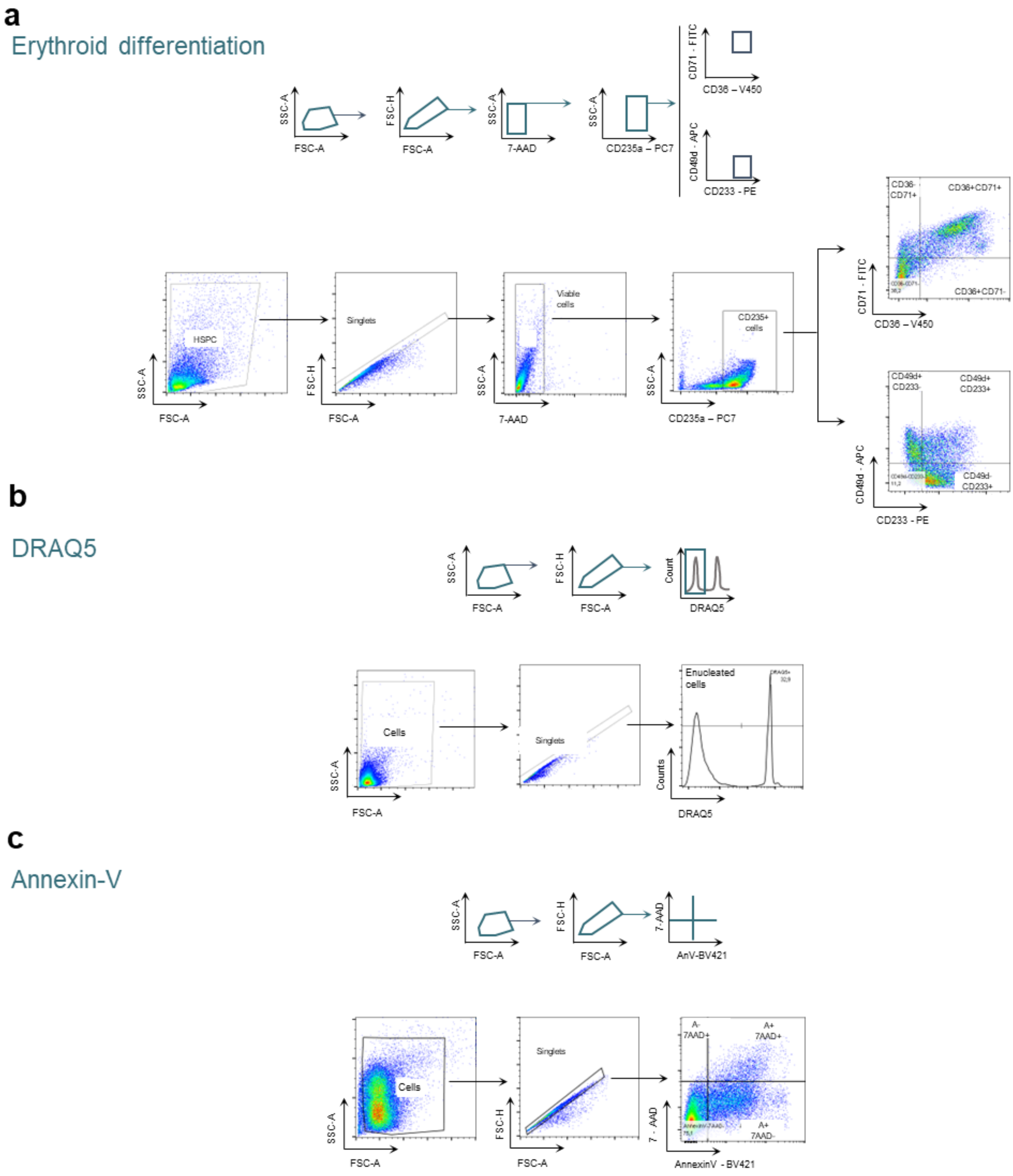

Supplementary Fig. 9: FCM analysis strategy for assessing in vitro erythroid differentiation of non-mobilized HSPCs derived from HbSS patients a FCM gating strategy for assessing selected markers during erythroid differentiation with representative FCM plots and analysis showing CD36+/CD71+ gating and CD233+/CD49d- gating. **b** FCM gating strategy for assessing DRAQ5- cells with representative FCM plots and analysis showing enucleated cells. **c** FCM gating strategy for assessing Annexin V-7AAD staining to detect live

(Annexin V-/7AAD-), early apoptotic (Annexin V +/7AAD-) and late apoptotic (Annexin V+/7AAD+) cells.

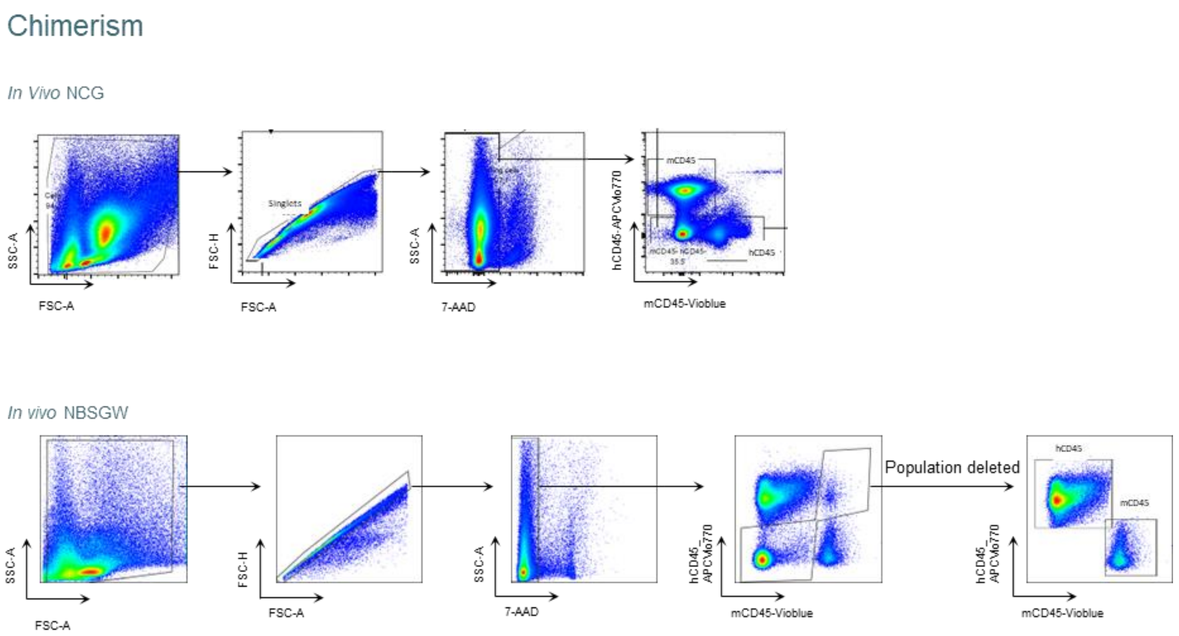

Supplementary Fig. 10: FCM analysis strategy for dissecting the engrafted human population in the bone marrow of NCG and NBSGW mice. Human CD45+ cells and murine CD45- cells were gated from the mononuclear cell suspension harvested from bone marrow at 16-18 weeks after injection in NCG or NBSGW mice. FCM gating strategy for deleting the CD45- population and assessing human chimerism (hCD45+/mCD45-). Representative FCM plot and analysis are shown for both murine models.

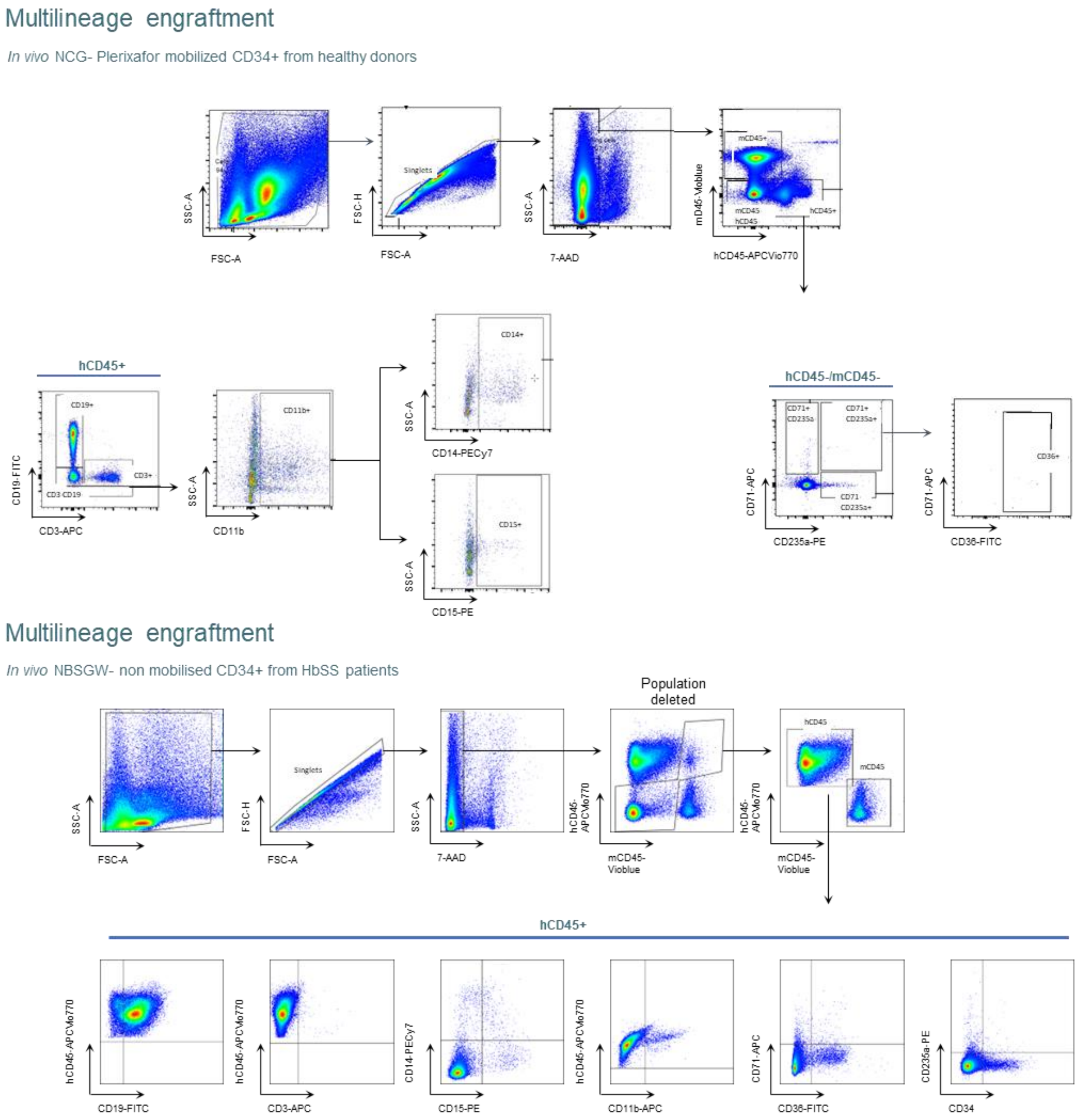

Supplementary Fig. 11: FCM analysis strategy for dissecting the multilineage engraftment in the bone marrow of NCG and NBSGW mice. Human CD45+ cells gated from the mononuclear cell suspension harvested from bone marrow at 16-18 weeks after injection in NCG or NBSGW mice were analyzed for hematopoietic lineage-specific markers for B cells (CD19+), T cells (CD3+), myeloid (CD14+, CD15+, CD11b+), and HSPCs (CD34+). Double negative cells were analyzed for the erythroid marker GPA (CD235a). FCM gating strategy for assessing multilineage engraftment with representative FCM plot and analysis are represented for both murine models.

Supplementary Tables

		Binding sequence		
**Talens**	**Target**	**Left Arm**	**Spacer**	**Right Arm**
TALEN-HBB$_{ss}$	SCD *HBB*	TCCTGTGGAGAAGTCTG	CCGTTACTGCC	CTGTGGGGCAAGGTGAA
TALEN-HBB$_{\beta\beta}$	WT *HBB*	TCCTGAGGAGAAGTCTG	CCGTTACTGCC	CTGTGGGGCAAGGTGAA

Correction Matrix	Sequence
ssODN	A*A*AAGTCAGGGCAGAGCCATCTATTGCTTACATTTGCTTCTGACACAACTGT<br>GTTCACTAGCAACCTCAAACAGACACCATGGTGCACCTGACACCAGAAGAGA<br>AGTCAGCCGTTACTGCCCTGTGGGGCAAGGTGAACGTGGATGAAGTTGGTGG<br>TGAGGCCCTGGGCAGGTTGGTATCAAGGTTACAAGACAG*G*T
AAV	GTTTTTAGTAGCAATTTGTACTGATGGTATGGGGCCAAGAGATATATCTTAGA<br>GGGAGGGCTGAGGGTTTGAAGTCCAACTCCTAAGCCAGTGCCAGAAGAGCCA<br>AGGACAGGTACGGCTGTCATCACTTAGACCTCACCCTGTGGAGCCACACCCTA<br>GGGTTGGCCAATCTACTCCCAGGAGCAGGGAGGGCAGGAGCCAGGGCTGGG<br>CATAAAAGTCAGGGCAGAGCCATCTATTGCTTACATTTGCTTCTGACACAACT<br>GTGTTCACTAGCAACCTCAAACAGACACCATGGTGCATCTGACACCAGAAGA<br>GAAGTCAGCCGTTACTGCCCTGTGGGGCAAGGTGAACGTGGATGAAGTTGGT<br>GGTGAGGCCCTGGGCAGGTTGGTATCAAGGTTACAAGACAGGTTTAAGGAGA<br>CCAATAGAAACTGGGCATGTGGAGACAGAGAAGACTCTTGGGTTTCTGATAG<br>GCACTGACTCTCTCTGCCTATTGGTCTATTTTCCCACCCTTAGGCTGCTGGTGGT<br>CTACCCTTGGACCCAGAGGTTCTTTGAGTCCTTTGGGGATCTGTCCACTCCTGA<br>TGCTGTTATGGGCAACCCTAAGGTGAAGGCTCATGGCAAGA

mRNA	Sequence
HDR-Enh (i53)	ATGTTGATTTTCGTGAAAACCCTTACCGGGAAAACCATCACCCTCGAGGTTGA<br>ACCCTCGGATACGATAGAAAATGTAAAGGCCAAGATCCAGGATAAGGAAGG<br>AATTCCTCCTGATCAGCAGAGACTGGCCTTTGCTGGCAAATCGCTGGAAGATG<br>GACGTACTTTGTCTGACTACAATATTCTAAAGGACTCTAAACTTCATCCTCTGT<br>TGAGACTTCGTTGA
Via-Enh (BCLXL)	ATGTCTCAGAGCAACCGGGAGCTGGTGGTTGACTTTCTCTCCTACAAGCTTTCC<br>CAGAAAGGATACAGCTGGAGTCAGTTTAGTGATGTGGAAGAGAACAGGACT<br>GAGGCCCCAGAAGGGACTGAATCGGAGATGGAGACCCCCAGTGCCATCAAT<br>GGCAACCCATCCTGGCACCTGGCAGACAGCCCCGCGGTGAATGGAGCCACTG<br>GCCACAGCAGCAGTTTGGATGCCCGGGAGGTGATCCCCATGGCAGCAGTAAA<br>GCAAGCGCTGAGGGAGGCAGGCGACGAGTTTGAACTGCGGTACCGGCGGGC<br>ATTCAGTGACCTGACATCCCAGCTCCACATCACCCCAGGGACAGCATATCAGA<br>GCTTTGAACAGGTAGTGAATGAACTCTTCCGGGATGGGGTAAACTGGGGTCG<br>CATTGTGGCCTTTTTCTCCTTCGGCGGGGCACTGTGCGTGGAAAGCGTAGACA<br>AGGAGATGCAGGTATTGGTGAGTCGGATCGCAGCTTGGATGGCCACTTACCTG<br>AATGACCACCTAGAGCCTTGGATCCAGGAGAACGGCGGCTGGGATACTTTTGT<br>GGAACTCTATGGGAACAATGCAGCAGCCGAGAGCCGAAAGGGCCAGGAACG<br>CTTCAACCGCTGGTTCCTGACGGGCATGACTGTGGCCGGCGTGGTTCTGCTGG<br>GCTCACTCTTCAGTCGGAAATGA

Supplementary Table 1

Detailed sequences for TALENs, HDR correction templates for ssODN and AAV and for mRNAs HDR-ENh01 and Via-Enh01.

Event detected	Primer / Probe	Sequence	Dye	Supplier	Note
HDR and NHEJ	Primer forward	CCAGGGCTGGGCATAAAAGT	-	IDT DNA	
	Primer reverse	CCAGGCCATCACTAAAGGCA	-	IDT DNA	
	Reference probe	CAAAGGACTCAAAGAACCTCT	FAM	IDT DNA	
	NHEJ probe	ACTGCCCTGTGGGGCAA	HEX	IDT DNA	
	HDR probe	CACCAGAAGAGAAGTCAGC	FAM	IDT DNA	
Inversion	Primer forward	TACGGCTGTCATCACTTAGACC	-	IDT DNA	
	Primer reverse	AGCCAATCTCAGGGCAAGTTAAG	-	IDT DNA	
	Probe	CCTAGGGTTGGCCAATCTACTCCC	FAM	IDT DNA	
Translocation	Primer forward	TACGGCTGTCATCACTTAGACC	-	IDT DNA	
	Primer reverse	GTAATCTGAGGGTAGGAAAACAGC	-	IDT DNA	
	Probe	CCTAGGGTTGGCCAATCTACTCCC	FAM	IDT DNA	
Deletion	Primer forward	AGCCAATCTCAGGGCAAGTTAAG	-	IDT DNA	
	Primer reverse	AGCCTAAGGGTGGGAAAATAGAC	-	IDT DNA	
	Probe	TCTTAAACCAACCTGCTCACTGGA	FAM	IDT DNA	
RPP30 control	Primer forward	AGTCTGTCGATGGTGTGCAG	-	IDT DNA	
	Primer reverse	GGAGGACATTTGAGGAGTGGT	-	IDT DNA	
	Probe	CAGTGGTGTGAGGGAGTTATCACT	HEX	IDT DNA	
Deepseq	HBB primer forward	CTACACGACGCTCTTCCGATCTCCAGGGCTGGGCATAAAAGT	-	IDT DNA	
	HBB primer reverse	GTGACTGGAGTTCAGACGTGTGCTCTTCCGATCTAATCCAGGCCATCACTAAAGGCA	-	IDT DNA	
CAST-SEQ	HBB_Bait-3	CCACATGCCCAGTTTCTATTG	-	IDT DNA	
	HBB_PCR1-1	GGTCTCCTTAAACCTGTCTTG	-	IDT DNA	
	Decoy_R-2	GAACACAGTTGTGTCAGAAGC	-	IDT DNA	
OCA	dsODN Forward	N*N*GTTTAATTGAGTTGTCATATGTTAATAACGGT*A*T	-	IDT DNA	* indicates a phosphorothioate linkage
	dsODN reverse	N*N*ATACCGTTATTAACATATGACAACTCAATTAA*A*C	-	IDT DNA	* indicates a phosphorothioate linkage
	P5_1	AATGATACGGCGACCACCGAGATCTA	-	IDT DNA	
	P5_2	AATGATACGGCGACCACCGAGATCTACAC	-	IDT DNA	
	P7	CAAGCAGAAGACGGCATACGAGAT	-	IDT DNA	
	dsODN-specific primer Forward (1st PCR)	ATACCGTTATTAACATATGACA	-	IDT DNA	
	dsODN-specific primer Forward (2st PCR)	CATATGACAACTCAATTAAAC	-	IDT DNA	
	dsODN-specific primer Reverse (1st PCR)	GTTTAATTGAGTTGTCATATGTTAATAAC	-	IDT DNA	
	dsODN-specific primer Reverse (2st PCR)	TTGAGTTGTCATATGTTAATAACGGTA	-	IDT DNA	
InferCNV	CRISPR TRAC	TGTGCTAGACATGAGGTCTATGG	-	IDT RNA	

Supplementary Table 2

Detailed sequences for primers and probes used for ddPCR, CAST-SEQ, OCA and AmpliconSeq.
