# Supplementary material for: Non-viral DNA delivery and TALEN editing correct the sickle cell mutation in hematopoietic stem cells
Source: Nat Commun. 2024 Jun 11;15:4965. doi: 10.1038/s41467-024-49353-3 (PMC11166989; doi:10.1038/s41467-024-49353-3)
Supplement: Supplementary file 3 — Reporting summary [file 41467_2024_49353_MOESM3_ESM.pdf]

nature portfolio

Corresponding author(s): Julien Valton
Arianna Moiani

Last updated by author(s): 2024_04_12

Reporting Summary

Nature Portfolio wishes to improve the reproducibility of the work that we publish. This form provides structure for consistency and transparency in reporting. For further information on Nature Portfolio policies, see our Editorial Policies and the Editorial Policy Checklist.

Statistics

For all statistical analyses, confirm that the following items are present in the figure legend, table legend, main text, or Methods section.

n/a	Confirmed	
☐	☒	The exact sample size (*n*) for each experimental group/condition, given as a discrete number and unit of measurement
☐	☒	A statement on whether measurements were taken from distinct samples or whether the same sample was measured repeatedly
☐	☒	The statistical test(s) used AND whether they are one- or two-sided<br>*Only common tests should be described solely by name; describe more complex techniques in the Methods section.*
☒	☐	A description of all covariates tested
☒	☐	A description of any assumptions or corrections, such as tests of normality and adjustment for multiple comparisons
☐	☒	A full description of the statistical parameters including central tendency (e.g. means) or other basic estimates (e.g. regression coefficient) AND variation (e.g. standard deviation) or associated estimates of uncertainty (e.g. confidence intervals)
☐	☒	For null hypothesis testing, the test statistic (e.g. F, t, r) with confidence intervals, effect sizes, degrees of freedom and P value noted<br>*Give P values as exact values whenever suitable.*
☒	☐	For Bayesian analysis, information on the choice of priors and Markov chain Monte Carlo settings
☒	☐	For hierarchical and complex designs, identification of the appropriate level for tests and full reporting of outcomes
☒	☐	Estimates of effect sizes (e.g. Cohen's *d*, Pearson's *r*), indicating how they were calculated

Our web collection on statistics for biologists contains articles on many of the points above.

Software and code

Policy information about availability of computer code

Data collection	Flow cytometry data were aquired MACSQuant 10 (Milteniy Biotech), BD CANTO (Becton Dickinson Bioscience), Fortessa X20 (Becton Dickinson Bioscience) or Gallios (Beckman coulter) flow cytometers. For measuring cell viability and concentration, data were aquired using NucleoCounter® NC-250. Single cell formulation was obtained using Chromium single cell system (10X Genomics), RNA Seq Hight throughput DNA sequencing was performed using MiSeq, NextSeq or NovaSeq systems (Illumina). HPLC experiment were performed using Nexera X2 HPLC system equiped with SIL-30AC autosampler. CFU colonies were detected and quantified using a STEMvision apparatus (STEMCELL Technologies). DNA quantification was performed using a NanodropOne device (ThermoFisher). ddPCR sample were prepared using QX200 Droplet Generator and analyzed using QX200 Droplet Reader (Bio-Rad). Transfection of HSPCs and T-cell was performed using the PulseAgile apparatus (Cellectis).
Data analysis	Macsquantify software v2.11 (Milteniy Biotech), BD FACS DIVA software v9.0 (BD), FLowJo software, FlowJo V.10.8.1 (Treestar), Prism software v9.2.0 (GraphPad). Cell viability was assessed using NucleoView™ software v4.3. DNA quantification was analyzed by NanoDrop QC software (ThermoScientific) v2.9.0.7. ddPCR were analyzed by QuantaSoft™ Software V1.7 (Bio-Rad). scRNA Seq sequencing read were demultiplexed and aligned to the human reference genome (GRCh38), using the CellRanger pipeline v6.1.2 (10X Genomics) and Azimuth was used to identify cell subpopulations. HPLC chromatograms were analyzed using LC solution software v5.51 (Shimadzu). CFU colonies were analyzed using STEMvision Colony marker (STEMCell technologies) v2.0.3.0.

For manuscripts utilizing custom algorithms or software that are central to the research but not yet described in published literature, software must be made available to editors and reviewers. We strongly encourage code deposition in a community repository (e.g. GitHub). See the Nature Portfolio guidelines for submitting code & software for further information.

Data

Policy information about availability of data

All manuscripts must include a data availability statement. This statement should provide the following information, where applicable:

- Accession codes, unique identifiers, or web links for publicly available datasets
- A description of any restrictions on data availability
- For clinical datasets or third party data, please ensure that the statement adheres to our policy

The authors declare that the data supporting the findings of this study are available in the article and in the supplementary information files. Source data are provided with this paper. The raw single cell transcriptomic datasets are available upon request.

Research involving human participants, their data, or biological material

Policy information about studies with human participants or human data. See also policy information about sex, gender (identity/presentation), and sexual orientation and race, ethnicity and racism.

Reporting on sex and gender	Sickle cell disease patients from the "Hôpital Necker-Enfants malades" Hospital (Paris, France) who were used as CD34+ HSPCs donors, remained anonymous to Cellectis. Their age, sex and gender were kept confidential by the "Hôpital Necker-Enfants malades" Hospital. This information was not collected by Cellectis.
Reporting on race, ethnicity, or other socially relevant groupings	Sickle cell disease patients from the "Hôpital Necker-Enfants malades" Hospital (Paris, France) who were used as CD34+ HSPCs donors, remained anonymous to Cellectis. Their race, ethnicity or other social relevant grouping were kept confidential by the "Hôpital Necker-Enfants malades" Hospital. This information was not collected by Cellectis.
Population characteristics	Sickle cell disease patients from the "Hôpital Necker-Enfants malades" Hospital (Paris, France) were used as CD34+ HSPCs donors. Their population characteristics were kept confidential by the "Hôpital Necker-Enfants malades" Hospital. This information was not collected by Cellectis. Thus, covariate-relevant population characteristics can't be reported.
Recruitment	Recrutment of HbSS patients was performed by the "Hôpital Necker-Enfants malades" Hospital (Paris,France). Frozen CD34+ HSPCs purified from healthy donor G-CSF-mobilized and Plerixafor-mobilized peripheral blood were purchased from AllCells (Almeda) or Hemacare (Los Angeles).
Ethics oversight	Frozen CD34+ HSPCs purified from healthy donor G-CSF-mobilized and Plerixafor-mobilized peripheral blood were purchased from AllCells (Almeda) or Hemacare (Los Angeles). CD34+ HSPCs derived from HbSS patients were recovered from erythrocytapheresis bags provided by the "Hôpital Necker-Enfants malades" Hospital (Paris, France). Written informed consent was obtained from all adult patients. The study was approved by the regional investigational review board (reference: DC 2022-5364, CPP Ile-de-France II "Hôpital Necker-Enfants malades").

Note that full information on the approval of the study protocol must also be provided in the manuscript.

Field-specific reporting

Please select the one below that is the best fit for your research. If you are not sure, read the appropriate sections before making your selection.

☒ Life sciences ☐ Behavioural & social sciences ☐ Ecological, evolutionary & environmental sciences

For a reference copy of the document with all sections, see nature.com/documents/nr-reporting-summary-flat.pdf

Life sciences study design

All studies must disclose on these points even when the disclosure is negative.

Sample size	For experiment other than in vivo studies, analyses were performed on at least 3 independently obtained cell batches. No calculation was performed to predetermine sample size. Sample size was determined from analogous studies performed by us and others where the differences between groups were expected to be comparable and the same statistical methods could be applied.<br>For animal studies, sample size was determined by power analysis (Charan, J. & Biswas, T. Indian J Psychol Med 35, 121-126, 2013) and corroborated by numerous comparable published studies using equal number or fewer animals per groups.
Data exclusions	No data were excluded from the analysis
Replication	The observations concerning the characterization of engineered HSPCs were replicated by repeating the experiments at lease three times, on HSPCs batches obtained as independently as possible from different healthy donors or HbSS patients. In general, HSPC batches were obtained by procedures performed at different times (at least a week apart) using shared reagents and most of the time by the same two operators. In some cases, different donors were engineered in parallel in the same procedures using the same batches of reagents. In less than 10% of the time, experiments failed to produce the desired cells to perform further analytical characterization (due to low transduction/transfection efficiency or poor viability of the cells). We confirm that once a sufficient number of viable cells were generated from a given donor with the

	expected engineered characteristics, all replication attempts were successful.
Randomization	For in vitro studies, we didn't considered covariates (other than the donor anonymous#) since HSPCs obtained from healthy donor and HbSS patients were fully anonymous (no record of sex, age, gender, ethnicity, race or social background were recovered and documented). For animal in vivo studies, mice were not distributed randomly into different experimental groups. Bodyweight was measured for all mice and groups were determined to minimize the differences in average bodyweight between groups. Experiment performed in vitro did not include randomization.
Blinding	Blinding was not performed. The analyses were objective measures not subject to bias.

Behavioural & social sciences study design

All studies must disclose on these points even when the disclosure is negative.

Study description	*Briefly describe the study type including whether data are quantitative, qualitative, or mixed-methods (e.g. qualitative cross-sectional, quantitative experimental, mixed-methods case study).*
Research sample	*State the research sample (e.g. Harvard university undergraduates, villagers in rural India) and provide relevant demographic information (e.g. age, sex) and indicate whether the sample is representative. Provide a rationale for the study sample chosen. For studies involving existing datasets, please describe the dataset and source.*
Sampling strategy	*Describe the sampling procedure (e.g. random, snowball, stratified, convenience). Describe the statistical methods that were used to predetermine sample size OR if no sample-size calculation was performed, describe how sample sizes were chosen and provide a rationale for why these sample sizes are sufficient. For qualitative data, please indicate whether data saturation was considered, and what criteria were used to decide that no further sampling was needed.*
Data collection	*Provide details about the data collection procedure, including the instruments or devices used to record the data (e.g. pen and paper, computer, eye tracker, video or audio equipment) whether anyone was present besides the participant(s) and the researcher, and whether the researcher was blind to experimental condition and/or the study hypothesis during data collection.*
Timing	*Indicate the start and stop dates of data collection. If there is a gap between collection periods, state the dates for each sample cohort.*
Data exclusions	*If no data were excluded from the analyses, state so OR if data were excluded, provide the exact number of exclusions and the rationale behind them, indicating whether exclusion criteria were pre-established.*
Non-participation	*State how many participants dropped out/declined participation and the reason(s) given OR provide response rate OR state that no participants dropped out/declined participation.*
Randomization	*If participants were not allocated into experimental groups, state so OR describe how participants were allocated to groups, and if allocation was not random, describe how covariates were controlled.*

Ecological, evolutionary & environmental sciences study design

All studies must disclose on these points even when the disclosure is negative.

Study description	*Briefly describe the study. For quantitative data include treatment factors and interactions, design structure (e.g. factorial, nested, hierarchical), nature and number of experimental units and replicates.*
Research sample	*Describe the research sample (e.g. a group of tagged Passer domesticus, all Stenocereus thurberi within Organ Pipe Cactus National Monument), and provide a rationale for the sample choice. When relevant, describe the organism taxa, source, sex, age range and any manipulations. State what population the sample is meant to represent when applicable. For studies involving existing datasets, describe the data and its source.*
Sampling strategy	*Note the sampling procedure. Describe the statistical methods that were used to predetermine sample size OR if no sample-size calculation was performed, describe how sample sizes were chosen and provide a rationale for why these sample sizes are sufficient.*
Data collection	*Describe the data collection procedure, including who recorded the data and how.*
Timing and spatial scale	*Indicate the start and stop dates of data collection, noting the frequency and periodicity of sampling and providing a rationale for these choices. If there is a gap between collection periods, state the dates for each sample cohort. Specify the spatial scale from which the data are taken*
Data exclusions	*If no data were excluded from the analyses, state so OR if data were excluded, describe the exclusions and the rationale behind them, indicating whether exclusion criteria were pre-established.*
Reproducibility	*Describe the measures taken to verify the reproducibility of experimental findings. For each experiment, note whether any attempts to repeat the experiment failed OR state that all attempts to repeat the experiment were successful.*

Randomization | *Describe how samples/organisms/participants were allocated into groups. If allocation was not random, describe how covariates were controlled. If this is not relevant to your study, explain why.*

Blinding | *Describe the extent of blinding used during data acquisition and analysis. If blinding was not possible, describe why OR explain why blinding was not relevant to your study.*

Did the study involve field work? ☐ Yes ☐ No

Field work, collection and transport

Field conditions | *Describe the study conditions for field work, providing relevant parameters (e.g. temperature, rainfall).*

Location | *State the location of the sampling or experiment, providing relevant parameters (e.g. latitude and longitude, elevation, water depth).*

Access & import/export | *Describe the efforts you have made to access habitats and to collect and import/export your samples in a responsible manner and in compliance with local, national and international laws, noting any permits that were obtained (give the name of the issuing authority, the date of issue, and any identifying information).*

Disturbance | *Describe any disturbance caused by the study and how it was minimized.*

Reporting for specific materials, systems and methods

We require information from authors about some types of materials, experimental systems and methods used in many studies. Here, indicate whether each material, system or method listed is relevant to your study. If you are not sure if a list item applies to your research, read the appropriate section before selecting a response.

Materials & experimental systems

n/a	Involved in the study
☐	☒ Antibodies
☒	☐ Eukaryotic cell lines
☒	☐ Palaeontology and archaeology
☐	☒ Animals and other organisms
☒	☐ Clinical data
☒	☐ Dual use research of concern
☒	☐ Plants

Methods

n/a	Involved in the study
☒	☐ ChIP-seq
☐	☒ Flow cytometry
☒	☐ MRI-based neuroimaging

Antibodies

Antibodies used | The list below document the antibody used in this study using the following typesetting: "Antibody-Fluorophore, Clone#, Dilution Manufacturer Catalogue#"
Human CD34-VioBlue, Clone#REA1164, 1/50 (Miltenyi, # 130-124-459)
Viability marker e780 1/1000 (eBioscience, #65-0865-18)
Human CD36-V450, Clone#CB38, 1/20 (BD, #561535)
Human CD71-FITC, Clone#M-A712, 1/50 (BD, #555536)
Human CD233-PE, Clone#BRIC 6, 1/50 (IBGRL, #9439)
Human CD235a-PECy7, Clone#GA-R2, 1/100 (BD, #563666)
Human CD49d-APC, Clone#9F10, 1/20 (BD, #559881)
Human Draq5, 1/500 (eBioscience, #65-0880-96)
Mouse CD45-Vioblue, Clone#REA737, 1/50 (Miltenyi, #130-110-664)
Human CD45-APCVio770, Clone# REA747, 1/50 (Miltenyi, #130-110-635)
Viability dye 7AAD, 1/200 (BD, #559925)
Human CD19-FITC, Clone#J3-119, 1/100 (Beckman Coulter, #A07768)
Human CD3-APC, Clone#REA613, 1/50 (Miltenyi, #130-113-135)
Human CD15-PE, Clone#80H5, 1/50 (Beckman Coulter, #IM1954U)
Human CD14-PECy7, Clone#MϕP9, 1/50 (BD, #562698)
Human CD11b-APC, Clone#M1, 1/100 (BD, #553312)
Human CD36-FITC, Clone#CB38, 1/50 (BD, #555454)
Human CD235a-PE, Clone#GA-R2, 1/50 (Invitrogen, #12-9987-82)
Human CD71-APC, Clone#M-A712, 1/50 (BD, #551374)

Validation | Commercial antibody validations were performed by suppliers. Validation of each antibody could be be found using the links below:
Human CD34-VioBlue, Clone#REA1164, 1/50 (Miltenyi, #130-124-459) https://www.miltenyibiotec.com/FR-en/products/cd34-antibody-anti-human-reafinity-rea1164.html#conjugate=vioblue:size=100-tests-in-200-ul
Viability marker e780 1/1000 (eBioscience, #65-0865-18) https://www.thermofisher.com/order/catalog/product/65-0865-18?

SID=srch-hj-65-0865-18
Human CD36-V450, Clone#CB38, 1/20 (BD, #561535) https://www.bdbiosciences.com/en-eu/products/reagents/flow-cytometry-reagents/research-reagents/single-color-antibodies-ruo/v450-mouse-anti-human-cd36.561535
Human CD71-FITC, Clone#M-A712, 1/50 (BD, #555536) https://www.bdbiosciences.com/en-eu/products/reagents/flow-cytometry-reagents/research-reagents/single-color-antibodies-ruo/fitc-mouse-anti-human-cd71.555536
Human CD233-PE, Clone#BRIC 6, 1/50 (IBGRL, #9439) https://nhsbtdbe.blob.core.windows.net/umbraco-assets-corp/6596/bric-6.pdf
Human CD235a-PECy7, Clone#GA-R2, 1/100 (BD, #563666) https://www.bdbiosciences.com/en-eu/products/reagents/flow-cytometry-reagents/research-reagents/single-color-antibodies-ruo/pe-cy-7-mouse-anti-human-cd235a.563666
Human CD49d-APC, Clone#9F10, 1/20 (BD, #559881) https://www.bdbiosciences.com/en-eu/products/reagents/flow-cytometry-reagents/research-reagents/single-color-antibodies-ruo/apc-mouse-anti-human-cd49d.559881
Human Draq5, 1/500 (eBioscience, #65-0880-96) https://www.thermofisher.com/order/catalog/product/65-0880-96?SID=srch-hj-65-0880-96
Mouse CD45-Vioblue, Clone#REA737, 1/50 (Miltenyi, #130-110-664) https://www.miltenyibiotec.com/FR-en/products/cd45-antibody-anti-mouse-reafinity-rea737.html#conjugate=vioblue:size=150-ug-in-1-ml
Human CD45-APCVio770, Clone# REA747, 1/50 (Miltenyi, #130-110-635) https://www.miltenyibiotec.com/FR-en/products/cd45-antibody-anti-human-reafinity-rea747.html#conjugate=apc-vio-770:size=100-tests-in-200-ul
Viability dye 7AAD, 1/200 (BD, #559925) https://www.bdbiosciences.com/en-fr/products/reagents/flow-cytometry-reagents/research-reagents/single-color-antibodies-ruo/7-aad.559925
Human CD19-FITC, Clone#J3-119, 1/100 (Beckman Coulter, #A07768) https://www.beckman.fr/reagents/coulter-flow-cytometry/antibodies-and-kits/single-color-antibodies/cd19/a07768
Human CD3-APC, Clone#REA613, 1/50 (Miltenyi, #130-113-135) https://www.miltenyibiotec.com/FR-en/products/cd3-antibody-anti-human-reafinity-rea613.html#conjugate=apc:size=100-tests-in-200-ul
Human CD15-PE, Clone#80H5, 1/50 (Beckman Coulter, #IM1954U) https://www.beckman.fr/reagents/coulter-flow-cytometry/antibodies-and-kits/single-color-antibodies/cd15/im1954u
Human CD14-PECy7, Clone#MϕP9, 1/50 (BD, #562698) https://www.bdbiosciences.com/en-fr/products/reagents/flow-cytometry-reagents/research-reagents/single-color-antibodies-ruo/pe-cy-7-mouse-anti-human-cd14.562698
Human CD11b-APC, Clone#M1, 1/100 (BD, #553312) https://www.bdbiosciences.com/en-fr/products/reagents/flow-cytometry-reagents/research-reagents/single-color-antibodies-ruo/apc-rat-anti-cd11b.553312
Human CD36-FITC, Clone#CB38, 1/50 (BD, #555454) https://www.bdbiosciences.com/en-fr/products/reagents/flow-cytometry-reagents/research-reagents/single-color-antibodies-ruo/fitc-mouse-anti-human-cd36.555454
Human CD235a-PE, Clone#GA-R2, 1/50 (Invitrogen, #12-9987-82) https://www.thermofisher.com/antibody/product/CD235a-Glycophorin-A-Antibody-clone-HIR2-GA-R2-Monoclonal/12-9987-82

Eukaryotic cell lines

Policy information about cell lines and Sex and Gender in Research

Cell line source(s): *State the source of each cell line used and the sex of all primary cell lines and cells derived from human participants or vertebrate models.*

Authentication: *Describe the authentication procedures for each cell line used OR declare that none of the cell lines used were authenticated.*

Mycoplasma contamination: *Confirm that all cell lines tested negative for mycoplasma contamination OR describe the results of the testing for mycoplasma contamination OR declare that the cell lines were not tested for mycoplasma contamination.*

Commonly misidentified lines (See ICLAC register): *Name any commonly misidentified cell lines used in the study and provide a rationale for their use.*

Palaeontology and Archaeology

Specimen provenance: *Provide provenance information for specimens and describe permits that were obtained for the work (including the name of the issuing authority, the date of issue, and any identifying information). Permits should encompass collection and, where applicable, export.*

Specimen deposition: *Indicate where the specimens have been deposited to permit free access by other researchers.*

Dating methods: *If new dates are provided, describe how they were obtained (e.g. collection, storage, sample pretreatment and measurement), where they were obtained (i.e. lab name), the calibration program and the protocol for quality assurance OR state that no new dates are provided.*

☐ Tick this box to confirm that the raw and calibrated dates are available in the paper or in Supplementary Information.

Ethics oversight: *Identify the organization(s) that approved or provided guidance on the study protocol, OR state that no ethical approval or guidance was required and explain why not.*

Note that full information on the approval of the study protocol must also be provided in the manuscript.

Animals and other research organisms

Policy information about studies involving animals; ARRIVE guidelines recommended for reporting animal research, and Sex and Gender in Research

Laboratory animals	Two types of animal model were used in the study:<br>-NODPrkdcem26Cd52 Il2rgem26Cd22/NjuCrl (NCG) mice. 8-weeks-old mice were used for this study<br>-NOD.Cg-KitW-41JTyr +Prkdcscid Il2rgtm1Wjl/ThomJ (NBSGW) mice. 5-weeks-old mice were used for this study
Wild animals	No wild animal was used in this study.
Reporting on sex	Female mice were used for the two studies reported in this manuscript.
Field-collected samples	No field-collected samples were used in this study.
Ethics oversight	-Regarding experiments performed with NOD.Cg-KitW-41JTyr +Prkdcscid Il2rgtm1Wjl/ThomJ (NBSGW) mice, all experiments and procedures were performed at Imagine Institut, in compliance with the French Ministry of Agriculture's regulations on animal experiments and were approved by the regional Animal Care and Use Committee (APAFIS#2019061312202425_v4).<br>-Regarding experiments performed with NODPrkdcem26Cd52 Il2rgem26Cd22/NjuCrl (NCG) mice, the work was performed in a GLP/GMP compliant laboratory in accordance with TCS Standard Operating Procedures (SOP), but without the involvement of TCS's Quality Assurance Unit. All procedures described in this study were reviewed and approved by the local ethic committee (Comité d'éthique Local pour l'expérimentation animale – Genevois, CELEAG).<br>Mice were monitored daily for unexpected signs of distress. Body weight was measured once a week. Mice with a cumulative clinical score ≥ 7 were euthanized. For a mouse with a body weight loss > 20% associated to a clinical score < 6, the veterinarian was consulted. The decision to euthanize an animal in pain or having reached the ethical limit was at the sole decision of the veterinarian.

Note that full information on the approval of the study protocol must also be provided in the manuscript.

Clinical data

Policy information about clinical studies

All manuscripts should comply with the ICMJE guidelines for publication of clinical research and a completed CONSORT checklist must be included with all submissions.

Clinical trial registration	*Provide the trial registration number from ClinicalTrials.gov or an equivalent agency.*
Study protocol	*Note where the full trial protocol can be accessed OR if not available, explain why.*
Data collection	*Describe the settings and locales of data collection, noting the time periods of recruitment and data collection.*
Outcomes	*Describe how you pre-defined primary and secondary outcome measures and how you assessed these measures.*

Dual use research of concern

Policy information about dual use research of concern

Hazards

Could the accidental, deliberate or reckless misuse of agents or technologies generated in the work, or the application of information presented in the manuscript, pose a threat to:

No	Yes	
☐	☐	Public health
☐	☐	National security
☐	☐	Crops and/or livestock
☐	☐	Ecosystems
☐	☐	Any other significant area

Experiments of concern

Does the work involve any of these experiments of concern:

No	Yes	
☐	☐	Demonstrate how to render a vaccine ineffective
☐	☐	Confer resistance to therapeutically useful antibiotics or antiviral agents
☐	☐	Enhance the virulence of a pathogen or render a nonpathogen virulent
☐	☐	Increase transmissibility of a pathogen
☐	☐	Alter the host range of a pathogen
☐	☐	Enable evasion of diagnostic/detection modalities
☐	☐	Enable the weaponization of a biological agent or toxin
☐	☐	Any other potentially harmful combination of experiments and agents

Plants

Seed stocks	n.a
Novel plant genotypes	n.a
Authentication	n.a

ChIP-seq

Data deposition

☐ Confirm that both raw and final processed data have been deposited in a public database such as GEO.

☐ Confirm that you have deposited or provided access to graph files (e.g. BED files) for the called peaks.

Data access links<br>*May remain private before publication.*	*For "Initial submission" or "Revised version" documents, provide reviewer access links. For your "Final submission" document, provide a link to the deposited data.*
Files in database submission	*Provide a list of all files available in the database submission.*
Genome browser session<br>(e.g. UCSC)	*Provide a link to an anonymized genome browser session for "Initial submission" and "Revised version" documents only, to enable peer review. Write "no longer applicable" for "Final submission" documents.*

Methodology

Replicates	*Describe the experimental replicates, specifying number, type and replicate agreement.*
Sequencing depth	*Describe the sequencing depth for each experiment, providing the total number of reads, uniquely mapped reads, length of reads and whether they were paired- or single-end.*
Antibodies	*Describe the antibodies used for the ChIP-seq experiments; as applicable, provide supplier name, catalog number, clone name, and lot number.*
Peak calling parameters	*Specify the command line program and parameters used for read mapping and peak calling, including the ChIP, control and index files used.*
Data quality	*Describe the methods used to ensure data quality in full detail, including how many peaks are at FDR 5% and above 5-fold enrichment.*
Software	*Describe the software used to collect and analyze the ChIP-seq data. For custom code that has been deposited into a community repository, provide accession details.*

Flow Cytometry

Plots

Confirm that:

☒ The axis labels state the marker and fluorochrome used (e.g. CD4-FITC).

☒ The axis scales are clearly visible. Include numbers along axes only for bottom left plot of group (a 'group' is an analysis of identical markers).

☒ All plots are contour plots with outliers or pseudocolor plots.

☒ A numerical value for number of cells or percentage (with statistics) is provided.

Methodology

Sample preparation	-In general, in vitro cell culture stainings were performed on harvested and PBS-washed cells, using the relevant mix of antibodies in staining buffer (PBS, 0.5% BSA, 2mM EDTA). Cells were incubated for 15 minutes with staining reagents at 4°C, followed by washes in excess volume of staining buffer.<br><br>-Erythroid liquid cultured cells were analyzed for enucleation (DRAQ5) and erythroid markers (GYPA, CD36, CD71, BAND3, !4-Integrin).<br><br>-Murine cells (bone marrow, spleen, thymus and blood) were analyzed for chimerism (mouse and human CD45 expression) and lineage specific markers (CD3, CD11b, CD14, CD15, CD19, CD235, CD36, CD71). Bone marrow was flushed out from femur and tibia and passed through a cell strainer to obtain a single cell suspension before staining. Spleen and thymus were smashed and passed through a cell strainer to obtain a single cell suspension before staining. Blood was lysed for RBC before staining.
Instrument	Flow cytometry data were aquired using MACSQuant 10 (Milteniy Biotech), BD CANTO (Becton Dickinson Bioscience), Fortessa X20 (Becton Dickinson Bioscience) or Gallios (Beckman coulter) flow cytometers.
Software	Macsquantify software v2.11 (Milteniy Biotech), BD FACS DIVA software v9.0 (BD), FLowJo software, FlowJo V.10.8.1 (Treestar), were used for flow cytometry dataset analysis.
Cell population abundance	The method used to determine the abundance of the different cell populations is described in the figures 8, 9, 10, 11 of the supplementary materials section of the manuscript.
Gating strategy	Gating strategies are described in the figures 8, 9, 10, 11 of the supplementary materials section of the manuscript.

☒ Tick this box to confirm that a figure exemplifying the gating strategy is provided in the Supplementary Information.

Magnetic resonance imaging

Experimental design

Design type	*Indicate task or resting state; event-related or block design.*
Design specifications	*Specify the number of blocks, trials or experimental units per session and/or subject, and specify the length of each trial or block (if trials are blocked) and interval between trials.*
Behavioral performance measures	*State number and/or type of variables recorded (e.g. correct button press, response time) and what statistics were used to establish that the subjects were performing the task as expected (e.g. mean, range, and/or standard deviation across subjects).*

Acquisition

Imaging type(s)	*Specify: functional, structural, diffusion, perfusion.*
Field strength	*Specify in Tesla*
Sequence & imaging parameters	*Specify the pulse sequence type (gradient echo, spin echo, etc.), imaging type (EPI, spiral, etc.), field of view, matrix size, slice thickness, orientation and TE/TR/flip angle.*
Area of acquisition	*State whether a whole brain scan was used OR define the area of acquisition, describing how the region was determined.*

Diffusion MRI ☐ Used ☐ Not used

Preprocessing

Preprocessing software	*Provide detail on software version and revision number and on specific parameters (model/functions, brain extraction, segmentation, smoothing kernel size, etc.).*
Normalization	*If data were normalized/standardized, describe the approach(es): specify linear or non-linear and define image types used for transformation OR indicate that data were not normalized and explain rationale for lack of normalization.*
Normalization template	*Describe the template used for normalization/transformation, specifying subject space or group standardized space (e.g. original Talairach, MNI305, ICBM152) OR indicate that the data were not normalized.*
Noise and artifact removal	*Describe your procedure(s) for artifact and structured noise removal, specifying motion parameters, tissue signals and physiological signals (heart rate, respiration).*
Volume censoring	*Define your software and/or method and criteria for volume censoring, and state the extent of such censoring.*

Statistical modeling & inference

Model type and settings	*Specify type (mass univariate, multivariate, RSA, predictive, etc.) and describe essential details of the model at the first and second levels (e.g. fixed, random or mixed effects; drift or auto-correlation).*
Effect(s) tested	*Define precise effect in terms of the task or stimulus conditions instead of psychological concepts and indicate whether ANOVA or factorial designs were used.*

Specify type of analysis: ☐ Whole brain ☐ ROI-based ☐ Both

Statistic type for inference<br>(See Eklund et al. 2016)	*Specify voxel-wise or cluster-wise and report all relevant parameters for cluster-wise methods.*
Correction	*Describe the type of correction and how it is obtained for multiple comparisons (e.g. FWE, FDR, permutation or Monte Carlo).*

Models & analysis

n/a	Involved in the study
☐	☐ Functional and/or effective connectivity
☐	☐ Graph analysis
☐	☐ Multivariate modeling or predictive analysis

Functional and/or effective connectivity	*Report the measures of dependence used and the model details (e.g. Pearson correlation, partial correlation, mutual information).*
Graph analysis	*Report the dependent variable and connectivity measure, specifying weighted graph or binarized graph, subject- or group-level, and the global and/or node summaries used (e.g. clustering coefficient, efficiency, etc.).*
Multivariate modeling and predictive analysis	*Specify independent variables, features extraction and dimension reduction, model, training and evaluation metrics.*
